# Understanding Educational Gradients in Diabetes and Hypertension: Triangulating Survey, Pharmacy, and Biomarker Data from High School & Beyond

**DOI:** 10.1002/alz70860_102237

**Published:** 2025-12-23

**Authors:** Eric Grodsky, John Robert Warren, Chandra Muller, Jennifer J. Manly, Adam Brickman

**Affiliations:** ^1^ University of Wisconsin ‐ Madison, Madison, WI, USA; ^2^ University of Minnesota, Minneapolis, MN, USA; ^3^ University of Texas at Austin, Austin, TX, USA; ^4^ Taub Institute for Research on Alzheimer's Disease and the Aging Brain, Vagelos College of Physicians and Surgeons, Columbia University, New York, NY, USA; ^5^ Columbia University, New York, NY, USA

## Abstract

**Background:**

Educational gradients in health conditions are profound, but our understanding of how to address them is limited by the data available to us. In large‐scale population‐based research, we most frequently evaluate the relationship between educational attainment (or years of schooling) and either (a) *self‐reported* incidence of adverse health events or diagnoses of health conditions or (b) biomarkers of those diseases.

Self‐reports are limited, however, by inequities and access to care; people with limited access to care may not know about health problems or conditions (at least until they reach acute levels). This means that inequities in (for example) self‐reported hypertension conflate actual inequities in disease with inequities in awareness of that disease.

Relying solely on biomarkers presents a different challenge for understanding health inequities ─ mainly because biomarkers often reflect both disease incidence and disease treatment. For example, a person with normal A1C levels may have never had diabetes or they may have been effectively treated for diabetes.

Until now, we have not had ─ for large, population‐representative cohort studies ─ all three of (a) people's self‐reports of disease; (b) biomarkers of disease; and (c) pharmaceutical records that indicate disease treatment. How do inferences about educational inequities in disease ─ specifically hypertension and diabetes ─ change when we have full information about all three?

**Method:**

To address this question, we use new data from the U.S. High School & Beyond (HS&B:80) cohort study, which has followed a nationally representative sample of ∼25,500 people from high school in 1980 through midlife in 2021‐22. HS&B:80 data from the 1980s includes rich information about educational contexts, opportunities, and outcomes; early life socioeconomic and family circumstances; spatial location; and demographic group memberships. Data from 2021‐22 include all three of (a) self‐reports of hypertension and diabetes; (b) blood pressure and A1C levels; and (c) pharmacy records on treatments for both conditions.

The results of this study will inform our understanding of health equity, and either reinforce our confidence in the reliability and validity of self‐reported health conditions or qualify how we interpret them.

**Result:**

Nothing yet.

**Conclusion:**

Nothing yet.

